# Attention Detection in Virtual Environments Using EEG Signals: A Scoping Review

**DOI:** 10.3389/fphys.2021.727840

**Published:** 2021-11-23

**Authors:** Rhaíra Helena Caetano e Souza, Eduardo Lázaro Martins Naves

**Affiliations:** ^1^Assistive Technology Laboratory, Electrical Engineering Faculty, Federal University of Uberlândia, Uberlândia, Brazil; ^2^Federal Institute of Education, Science and Technology of Brasília, Brasília, Brazil

**Keywords:** attentional orientation, virtual reality, cognitive workload, fatigue, EEG signal

## Abstract

The competitive demand for attention is present in our daily lives, and the identification of neural processes in the EEG signals associated with the demand for specific attention can be useful to the individual’s interactions in virtual environments. Since EEG-based devices can be portable, non-invasive, and present high temporal resolution technology for recording neural signal, the interpretations of virtual systems user’s attention, fatigue and cognitive load based on parameters extracted from the EEG signal are relevant for several purposes, such as games, rehabilitation, and therapies. However, despite the large amount of studies on this subject, different methodological forms are highlighted and suggested in this work, relating virtual environments, demand of attention, workload and fatigue applications. In our summarization, we discuss controversies, current research gaps and future directions together with the background and final sections.

## Introduction

The study of EEG signals associated with the application in different technologies is a great interest in neuroscience, for example, in assistive technology/rehabilitation tools, as it is a non-invasive recorded physiological signal, with information on the individual’s conscious and unconscious states in reaction to external and internal stimuli. The external stimulus can be a virtual reality system (VR), composed of sound and, mainly, image, being a means of communication that allows the user to feel physically present from the virtual experience. VR systems can present different levels of immersion of the user in the virtual environment, the more immersive, the greater the feeling of belonging of the user and, the more consonant with the information generated in the EEG signal it will be. In this case, an encouraging, personalized interface with a reliable replication of a real environment is favorable. This allows the brain EEG signal to be extracted from an individual with exposure to a controlled scenario, similar to the real one but limited to the stimuli that reach their perception.

In virtual environments, the user is exposed to punctual and constant stimuli, some with greater intensity than others. Thus, levels of attention and engagement can change and when monitored with the help of equipment that records non-invasive electrophysiological neural signals (EEG), it is possible to infer the individual’s dedicated attention, modulated by intrinsic and extrinsic factors. There is a great current interest in detecting attention and engagement based on EEG signals, which are responses to the efficiency of an application with intense virtual stimulation, as we will exemplify throughout this work. Even in situations where competing stimuli are absent, consistent attention to the virtual system is a challenge. Therefore, the modulation of neural responses by states of attention has been widely explored, and this aspect, extrapolated from the EEG signal, is important to relate to the applied immersion system and understand the effectiveness of the EEG-based virtual system application.

This review aims, therefore, to present an updated overview of the approaches associated with the level of attention detected employing features extracted from the EEG signal with techniques applied in neuroscience (Table 1 and Supplementary Table 1), and how they currently relate to virtual reality systems. Future directions are extrapolated and presented in such a way that attention carries with it different mechanisms to dynamically adapt the extrinsic and intrinsic cognitive load to the human being; this characteristic, therefore, is capital in neuroscientific systems, which involve parallel processing of data, very common today.

**TABLE 1:** Brain waves and corresponding authors bands variations.

**Brain wave**	**Frequency band (Hz)**
**Delta (δ)**	**0.5–3.5 Hz** (Cowley and Ravaja, 2014); **0–3.9 Hz** (Hazarika et al., 2018); **0.1–3 Hz** (Coenen et al., 2020); **0–5 Hz** (Mathewson et al., 2012); **1–3 Hz** (Wang Y. K. et al., 2015); **1–4 Hz** (Wang Y. et al., 2020); **2–4 Hz** (Heyselaar et al., 2018)
**Theta (θ)**	**3–8 Hz** (Jaquess et al., 2017); **3.5–8 Hz** (Cowley and Ravaja, 2014); **3.9–7.8 Hz** (Hazarika et al., 2018); **4–8 Hz** (Wang Q. et al., 2011; Matthews et al., 2015; Vortmann et al., 2019); **3–10 Hz** (Savage et al., 2013); **4–7 Hz** (Clemente et al., 2014; Jagannath and Balasubramanian, 2014; Wang Y. K. et al., 2015; Wang Y. et al., 2020; Fuentes-García et al., 2019; Coenen et al., 2020; Li et al., 2020b); **5–8 Hz** (Heyselaar et al., 2018)
**Alpha (α)**	**7–12 Hz** (Mathewson et al., 2012); **7.8–15.6 Hz** (Hazarika et al., 2018); **8–12 Hz** (Clemente et al., 2014; Jagannath and Balasubramanian, 2014; Berger and Davelaar, 2018; Coenen et al., 2020; Wang Y. et al., 2020); **8–13 Hz** (Cowley and Ravaja, 2014; Wang Y. K. et al., 2015; Jaquess et al., 2017; Li et al., 2020b); **9–13 Hz** (Matthews et al., 2015); **8–14 Hz** (Heyselaar et al., 2018; Vortmann et al., 2019)
**Beta (β)**	**12–30 Hz** (Wang Q. et al., 2011); **13–25 Hz** (Coenen et al., 2020); **13–30 Hz** (Cowley and Ravaja, 2014; Jagannath and Balasubramanian, 2014; Jaquess et al., 2017; Li et al., 2020b; Wang Y. et al., 2020); **13–32 Hz** (Yin and Zhang, 2014); **14–30 Hz** (Matthews et al., 2015; Vortmann et al., 2019); **15–20 Hz** (Heyselaar et al., 2018); **15.6–31.25 Hz** (Hazarika et al., 2018)
**Gamma (γ)**	**25–50 Hz** (Coenen et al., 2020); **31.25–62.5 Hz** (Hazarika et al., 2018); **30–45 Hz** (Cowley and Ravaja, 2014; Vortmann et al., 2019); **33–40 Hz** (Yin and Zhang, 2014); **30–120 Hz** (Wang Y. et al., 2020)

*Brain waves frequency bands are indicated in bold.*

## Background

### Attention Allocation and Fatigue

In the study field of attention, several definitions are needed in order to interpret the results presented in scientific findings. William James once wrote in the principles of psychology that “Everyone knows what attention is” (James, 1891), but, paradoxically, attention is one of the most researched terms in neuroscience, biology and psychology, sciences that seek to classify objectively the mechanisms of attention and how it dynamically adapts to internal and external information flows. Thus, it is more convenient to describe that “Nobody knows what attention is,” as the authors explore more recently (Hommel et al., 2019; Lindsay, 2020). In a new and more current approach, authors such as Chun and Cho (Chun et al., 2011; Cho et al., 2015) have explored the concept of attention and its role in artificial neural networks, in the context of machine learning. Attention, therefore, is far from having a clear and unified conceptualization, but it can be understood in a general way as selective capacity to control limited resources of cognitive load processes.

Some of the taxonomies that involve the concept of attention need to be defined in the context of this work: covert and overt attention; bottom-up and top-down attention; feature and spatial attention. Covert and overt attention can be differentiated in relation to the observation of objects around, the first without specific visual focus by the individual, and the other detected in the observation of target objects, respectively. Bottom-up and top-down attention are different forms of classification of attention, in the first case an abrupt/spontaneous/involuntary stimulus (e.g., objects with strong color contrasts) draws attention from the external environment vs. a dedicated attention in the second case, one that was planned and voluntary (e.g., solving arithmetic calculations). Corbetta and Shulman (2002) define as goal-directed and stimulus-driven the top-down and bottom-up attention elements, respectively. Feature and spatial attention are differentiated as the name suggests, the first looking for specific characteristics (e.g., look for all yellow things) and the second for specific objects with specific qualities (e.g., looking for a yellow flower).

Above all, feature and spatial attention are associated with visual stimulation. In spatial attention, the saccade movement stands out in overt attention: rapid movement of the eyes between two fixation points. For the shift of the gaze focus to occur, the individual’s attention-calling stimulus will be easily identified by the result it causes in the final action: saccade. Although the external stimulus may have caused this abrupt change in the focus of the individual’s gaze, it is understood that the saccade movement corroborates a directed spatial visual attention, with a specific target object, being, therefore, an example of execution of overt attention. In covert attention, on the other hand, attention is oriented to a specific location, in which visual stimuli external to that fixation location must be identified by the individual, as a task, but must not cause a change of look or saccade movement. Covert and overt attention, then, can be understood by means of visual behavior.

Attention is strongly affected by sensory inputs such as vision and hearing, but there is a natural tendency in attention studies to emphasize results obtained from visual stimuli as discussed by Hutmacher (2019). This author considers that vision is socially and culturally dominant over other senses, which gives us room to reflect on technological approaches and the predominance of visual input stimuli in the orientation of the individual’s attention, as in the case of virtual reality systems. In a more recent approach, “free-viewing” experiments emerged in real and virtual environments, where the individual is not directed to any specific task, he is only inserted in the context of interest, reacting to the scenario in a contemplative way. Everyday tasks, such as watching children play in the pool, is a free-viewing fashion, which can provoke neural patterns detected in EEG cognitive processes with low mental effort. More recently, the attention state that has no specific focus, in a free-viewing fashion, was explored between two determined tasks and, as result, authors (Li et al., 2020a) found that this period of “mind wondering” possibly was influenced by the directions given for the task execution and by the stimuli of the task itself, even in a moment of a lack of task. Others known approaches are explored next.

**FIGURE 1:**
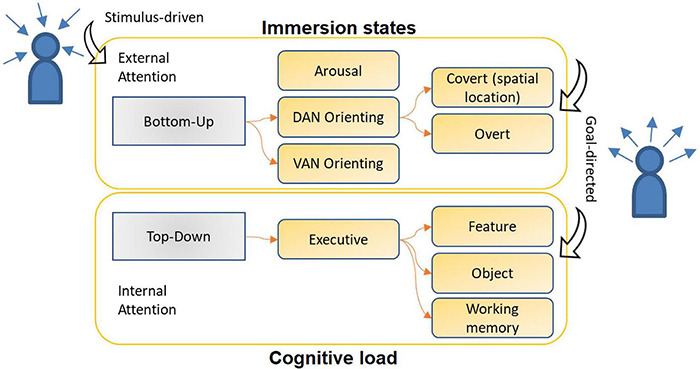
Attention taxonomy merging traditional definitions. Attention allocation can be detected from different approaches, the internal or external triggers may characterize attention in immersive or cognitive load state. Upper box: the arrows point to the subject’s head – the elements that make up the stimulus path come from the external environment into the individual – so the attention allocation is guided by the stimulus – ‘stimulus- driven’ – prevailing in higher immersion states (due to screen or virtual environment stimuli). ‘Arousal’ – first box and first reflex of the stimulus perception. Lower box: elements that involve internal attention allocation, ‘executive’ relates the first cognitive reflex of the content selection for oriented attention. ‘Goal-directed’ – attention oriented from an internal stimulus – prevailing in higher cognitive load states –, but, the goal-directed mechanism also occurs when external stimulus-oriented attention reaches its target, so the goal-directed arrows cross both stimuli directions showing the two boxes interaction. Schematic overview inspired on (Chun et al., 2011) work.

Petersen and Posner (2012) present in their work three functionally independent brain networks that are affected by attention: alertness/vigilance/arousal, orientation (processes stimuli from the external environment, there is a dorsal and a ventral network in the human body) and execution (complex and oriented tasks). Orienting and execution networks can be described according to Chun’s taxonomy (Chun et al., 2011) for external and internal attention, respectively. The dorsal network (DAN) is reflected in relation to involuntary attention in the superior parietal, occipital and frontal cortex electrodes. The ventral network (VAN) comprises the control of attention focus, voluntary attention, by releasing norepinephrine in various parts of the brain, including non-cortical regions, such as the anterior insula and temporoparietal junction, and cortical regions, such as the anterior cingulate cortex and pre-fronts. These two networks complement each other in the operation of oriented (internal) attention with external stimuli that provoke attentional reorientation (Proskovec et al., 2018). Figure 1 shows the different conceptualizations discussed, illustrating how they are complementary, sometimes overlapped.

The level of attention, level of alertness and awake state are confused in scientific research, referring to the same individual engagement skill in relation to a given context or artifact. Awake state, in turn, concerns how much this engagement can be sustained. Although the level of arousal is important in assessing the individual’s performance when performing tasks, it cannot be very high, causing unwanted results, as illustrated by the well-known Yerkes-Dodson curve, better discussed in section *B*. The level of attention/arousal was associated as far back in 1929 with records of oscillations in the alpha band (Berger, 1929). The decay in the alpha band and, consequently, its power associated with an increase in the delta band are very known indications of attentional engagement in individuals (Klimesch et al., 1998). Until today, the main findings of new studies when assessing the individual’s attention and relating it to the ability of cognitive control are associated with alpha waves, although with controversial results, as we will see at the end of this section.

It is common to study the sources that influence attention and not the multiple brain processing systems that can be affected by attention. Simultaneously, the human brain can involve two distinct processing areas, such as the auditory task and the visual task, sharing the individual’s attention (Posner et al., 1989; Petersen and Posner, 2012). More recently studies have highlighted the multiple simultaneous brain processing systems affected by concurrent attention (Long and Kuhl, 2018; Itthipuripat et al., 2019), as well as investigated how diverse media that require user multitasking affects attention and, consequently, cognitive load processes (Uncapher et al., 2017). We will deal more with the workload state related to multitasking in section *B*, but it is known that in multitasking scenarios, which require more attention, there is a change in neural activity, causing a higher firing rate of neurons, typically 20–30% more (Mitchell et al., 2007), which can be identified by the EEG signal with increasing brainwave bands.

Regarding brainwave bands, the increase in α – alpha waves (low alpha 8–11 Hz and high alpha 11.1–13 Hz) has been the main physiological indicator of low anxiety state, especially high alpha (Li et al., 2020c) which indicates also cognitive idleness, being a marker of external stimuli suppression capable of reorienting the individual’s attention (Vortmann et al., 2019). In this sense, there is an indicator of the alpha wave on the top-down attention direction, a restorative process that reduces anxiety and fatigue. As alpha, theta and delta waves increase proportionally in relation to beta and gamma waves, the level of alertness decreases (Trejo et al., 2015). The individual’s immersion in environments that encourage contact with nature, with the intention of transmitting tranquility, relaxation and increased meditation activity, increases the power of alpha waves (Grassini et al., 2019).

Several experiments have explored the condition of individuals in relaxing environments and its reflection on the EEG. In laboratory conditions, findings were reported with stimulation to natural environments that promote tranquility, increased alpha activity, relaxation, increased meditation power, among others (Grassini et al., 2019). In uncontrolled environments, with physical and natural exposure to nature, with the use of portable EEG systems, results corroborate those found with the use of artificial environments (Aspinall et al., 2015; Chen et al., 2016): greater engagement and arousal, less frustration and greater meditation. The attention restoration theory (ART) deals with the properties of natural environments that unintentionally attract the individual’s attention, eradicating anxiety and mental fatigue, expanding the performance capacity of the cognitive focus (Kaplan and Kaplan, 1989).

The qualities of natural environments are relevant and are treated by Kaplan (2001), according to ART. Four key components are decisive in natural environments: 1- the individual’s ability to be psychologically disconnected, as portrayed by the Psychological Recovery Theory (PRT), of present concerns and demands, is called “being away”; 2 – the ability to capture the individual’s attention with involuntary factors (bottom-up), generating the minimum of disturbances that drain voluntary attention (top-down), is called “soft fascination”; 3 – the level of immersion that the individual feels, being able to distinguish cognitive maps, and what is the engagement generated with this sensation, is called “extent”; 4 – the intrinsic motivation of the individual to remain in a certain environment, to feel part of or want to do what he wants, is called “compatibility.” The fascination state is very explored in literature.

Kaplan (1995) determines two definitions to distinguish fascination. The first is “hard fascination” and the second is “soft fascination,” the first covers abrupt and high stimuli so that the individual must act quickly, without reflecting on the stimulus, which can generate stress and mental fatigue. In the second, the individual is surrounded by a visually pleasing aspect, such as natural environments, allowing him to reflect on the event that calls his attention, promoting attention restoration. In the first, we can associate the definition of overt attention, in which attention is directed to the stimulus focus, from a quick movement of the eyes, for example, saccade. In the second, covert attention predominates, encompassing the aspect of free-viewing and natural contemplation of the elements around the individual.

In this context, the individual’s state of engagement, comprising the qualities pointed out by ART, is highlighted (Ohly et al., 2016), in order to enable benefits based on the dedication of their attention for some time in the contemplation of natural environments. How this occurs, and how the method can be replicated, based on quantitative EEG results, remains unclear. None of the studies cited on ART in a recent review (Ohly et al., 2016) reported neural data related to the attention restoration process. After the aforementioned review article, the authors Chung and Li (Chung et al., 2018; Li et al., 2020d) present in their work studies exploring ART and EEG neural signals. Both found variations in the evoked potential P3 in exposures to virtual environments with simulation of nature, in 5 and 30 min, respectively, for the cited studies. The states of relaxation and attention restoration involve a low level of arousal and alertness, however, they differ from the state of mental fatigue, which also presents these characteristics.

Mental fatigue is a mental state after an operation for a long period of high mental effort, when there is cognitive demand, tiredness sensation, sleep deprivation, unwillingness to new mental efforts and, mainly, compromise of the individual’s performance in tasks. Indicators of mental fatigue can be subjective and objective. In the subjective assessment, indicators such as headache, tiredness, inability to concentrate, among others, can be assessed via an individual questionnaire and exposed to a subjective bias. In the objective evaluation, through the EEG signal, a shift of EEG power toward low-frequency bands (δ,θ, and α) has been widely reported in the past (Wascher et al., 2014), and the increase of low alpha (8–11 Hz) waves is currently accepted associated with increased level of fatigue (Zou et al., 2015; Li et al., 2020c), but the opposite has been widely reported in the past (Jap et al., 2009). These oscillations are more identified in frontal, frontotemporal and over visual regions (Lamti et al., 2016), with high frequencies (beta, gamma) typically decaying in amplitude (Grassini et al., 2019). A decrease in the level of arousal, as well as a reduction in goal-directed attention, a reduction in effective selective attention and an increase in the difficulty of dividing attention into multitasking are aspects found as a result of mental fatigue states (Trejo et al., 2015; Lamti et al., 2016).

The main investigations to identify mental fatigue from the EEG signal are associated with driver monitoring when driving vehicles (real or virtual), by the direct implication of the results (Jagannath and Balasubramanian, 2014; Touryan et al., 2016). It is known that most car accidents are due to human errors and that they can be related to mental fatigue or lack of attention by the vehicle driver. Virtual wheelchair driving was assessed in relation to mental fatigue, by EEG signals, in the work of Lamti (Lamti et al., 2016). Detection of mental fatigue in the neurophysiological processes is widely reported through the EEG feature extraction (Li et al., 2020c), such as the delta and theta ratio with alpha and beta bands. However, few studies investigate the differences of mental fatigue in the processes of attention allocation, through the EEG activity, with virtual environment stimuli.

To contemplate the state of attention restoration, the lack of attention factor, associated or not with mental fatigue, must be previously identified. This affects individuals in their daily life, due to the feeling of tiredness, compromising the tasks performance that require mental effort. In section *B*, we will bring more details about what task performance is, how the workload, working memory and learning processes can be identified and can culminate in mental fatigue.

### Attentional Orientation and Cognitive Workload

The learning process and mental load measurement can be investigated on several fronts, such as attention, response time and task development (time on-task), expertise, self-regulation, and multimedia learning. The high workload information associated with the study of attention may be relevant to understand the impacts of brain signal variation in face of changes in engagement, mood and task difficulty. Successful of within task difficulty level classification has been happening for some years, but it remains unclear and challenging the classification of cross-talk (unwanted co-recorded EEG signal from multiple electrodes) (Nelson et al., 2017). There is also great interest in monitoring the mental workload of individuals in everyday situations, especially in cases where the high demand for mental workload can compromise attention, such as driving (Sumwalt et al., 2019). There is no consensus among the works mentioned above for the definition of mental workload, as several nomenclatures [mental workload (MWL), working memory load (WML), cognitive learning, mental load, task load and cognitive workload] surround aspects of cognitive load, but it can be understood in general as the task demand and the individual’s ability to perform in front of that task within a context (Young et al., 2015).

In the context of cognitive neuroscience, attention is one of the three cognitive control abilities (the other two are working memory and goal management) (Gazzaley and Rosen, 2016). The variables that characterize mental load are evaluated after long periods of the individual’s practical exposure to cognitive load stimuli, with attention control, which consists of not only successfully perform selected tasks, but also free practices to acquire experience, with enthusiasm (engagement in the face of stimuli that generate cognitive demand), top-down attention and perseverance for long-term goals, with no immediate result. A multitasking practice is always related, in research, to the amount of time needed to develop a certain skill, as discussed by Chase and Simon (1973) in their seminal article, but also to the engagement and arousal that the individual demonstrates in activity, which can commonly be measured by the response time (RT) of cognitive processing to the stimulus (Li et al., 2020b,d).

As well as relating the levels of arousal and cognitive performance in the most accepted and widespread theory of the Yerkes-Dodson law bell curve, it is observed that with the increase in the level of arousal, anxiety is generated and impairs the performance potential in the resolution of multiple activities or difficult activities. The weakest the individual’s performance is, the lowest is arousal in attention identification. A higher arousal can cause low performance in the presence of fatigue in the task. There is an optimal phase in the medium arousal and maximal performance, when engagement and attentional orientation are at its best. In the simulation of a complex task, such as flying an airplane, the high mental workload demanded impairs the ability of language processing, as well as the individual’s performance in the task (Causse et al., 2016). Analogous to the reasoning proposed in the curve, the increase in stress due to a high cognitive load or difficulty of a task follows the same pattern, generating cognitive impairment due to fatigue. The authors McKendrick et al. (2019) called the Stress-Strain curve the capacity model analogous to the Yerkes-Dodson curve, to demonstrate the behavior of the application of mental load in the states of individual capacities. The authors Li et al. (2020c) suggest, in the case of overload, an increase in the low alpha band (8–11 Hz), while the authors Gevins and Smith (2003) and Borghini et al. (2012) found that theta-band spectral power increased in the frontal midline and attenuation of the alpha band in general, perceived in the EEG signal.

Electrophysiological factors, such as the brain signal recorded through the EEG, can be measured objectively and quantitatively, and are associated with the learning rate of individuals in game manipulation. Complex video games have long been recognized as useful investigative techniques for cognitive neuroscience as well as potential cognitive training tools (Donchin, 1989). The authors Maclin and Mathewson (Maclin et al., 2011; Mathewson et al., 2012) have already reported the modulation of event related spectral perturbation (ERSP), detected by the EEG signal, in games without immersion as increasing the sources of attention for events of secondary tasks. Specifically, the ERSPs in the α (7–12 Hz) and δ (0–5 Hz) bands in a range of games were identified with change as learning increased. The increase in P300 amplitude in secondary tasks was shown to indicate that sources of attention were released as game manipulation became more automated. As described by Chang and Lin (2011), this occurs because the user’s expectation decreases the stimulus perception threshold, reducing the recorded potential latency.

Event-related potentials (ERPs) are brain electrical potentials, perceived in the EEG (such as the ERSP and P300 mentioned above), associated with the individual’s internal and external cognitive stimuli. Therefore, they can be the result of top-down or bottom-up attention, in terms of attentional orientation. ERPs can be the result of cognitive processes, but are also easily affected by mood and emotion, and are analyzed with a focus on time-domain monitoring of the EEG signal at a specific interval after the source stimulus. The trigger for the primary stimulus is a crucial factor in understanding these potentials, which are validated in terms of their amplitude and latency measures, and may be an intra- or inter-individual assessment. In general, scientific research fails to demonstrate the ERPs validation.

A variety of spectral characteristics/dynamic variables can be extracted from the EEG signal as indicative of cognitive variation, which provide satisfactory measures and classification of cognitive workload. The EEG signal features found in the investigated articles will be shown in the results of this work and most investigations of mental load are by means of the maximum or average power spectral density (PSD) (Yin and Zhang, 2014; Matthews et al., 2015; Yu et al., 2015; Magosso et al., 2019), entropy-based features such as the wavelet packet also show up (Stam et al., 2002; Zarjam et al., 2013). Other approaches refer to spectral characteristics coherence and Phase Locking Value (PLV), these synchronization events are of interest due to the brain firing state necessary for conducting many cognitive functions.

Event-Related Synchronization and Desynchronization (ERS and ERD, respectively) will be dependent on each task demand or stimulus type, and may be recognized in alpha power increase and decrease, respectively. ERD is associated with increased excitability of cortical neurons during cognitive neural processes, motor and sensory control (Neuper and Pfurtscheller, 2001), while ERS surrounds focal ERD, assisting in by increasing and synchronizing the neighboring cortical areas, where potentially interfering cues and distractors may occur (Pfurtscheller, 2003; Foxe and Snyder, 2011). However, the standardization and precision of mental workload measures in identifying different levels of difficulty are still unclear.

As more cognitive tasks are applied, the brain load decreases with the increase in cognitive inefficiency, consequently the power ratio of alpha and beta waves in the EEG signal (indicative of engagement) decreases in each brain region. The cognitive load of each individual will be perceived as adequate, high or low (adequate load, overload or underload) depending on their capacity. Three ways to measure cognitive load are: self-report (qualitative), behavioral secondary tasks and physiological measures (quantitative) (McKendrick et al., 2019). Cognitive load levels are usually modulated with arithmetic tasks or simulation tasks for some scenarios, such as driving simulation, air traffic control and games with avatars. In the latter case, the aid of scenario simulation requires great engagement, demands visual and spatial attention, bottom-up and top-down attention, and can be compared to the stimulus of real complex scenarios, which can also culminate in visual fatigue of the 3D environment.

In order to assess levels of cognitive load across a range of load situations in a qualitative and subjective way, the NASA Task Load Index Scale (NASA TLX) is the most widely used set to measure the workload through six sub-scales, each of which associated to a source of workload (mental demand, physical demand, temporal demand, performance, effort and frustration) (Morrison et al., 2014) and has become synonymous with the concept of mental workload (de Winter, 2014). Combining those sub-scales results in an overall score which the physical and cognitive load of the subject, can be helpful to separate between emotion and mental fatigue income. However, this assessment is subjective and is hostage to memory lapses as well as imprecision bias. This subjective investigation is often accompanied by an investigation of behavioral secondary tasks. Quantitative investigation via electrophysiological signals such as EEG is less susceptible to subjectivity bias and abrupt change in response.

### Electrophysiological Signals and Neuroscience

The EEG signal extracted from the scalp surface can be divided into frequency bands, which have stochastic characteristics of similar amplitude and frequency. The different bands of brain frequencies carry information about the states of individuals, as we present examples in sections A, B, and C. The amplitude of the EEG signal through the cerebral scalp is normally between 10 and 100 μV, the divisions in frequencies are: δ – delta band (less than 4 Hz), θ – theta band (4–8 Hz), α – alpha band (low alpha 8–11 Hz and high alpha 11–13 Hz) and β – beta band (13–30 Hz), are respectively associated with commonly recognized individual states: deep sleep, state of relaxation and meditation, state of awareness and relaxation and, finally, active thinking and higher cognitive load. These bands are the most applied in the analysis of EEG signals of low frequency, which may vary some Hertz among different authors, as shown in Table 1, with another possibility being the exploration of high frequency oscillations in the EEG signal (γ – gamma band, greater than 30 Hz), which are generally associated with some mental illnesses diagnosis.

The use of EEG-based devices has become popular with the advancement of EEG headsets, as it lowers the cost of capturing EEG signals and allows its use in uncontrolled environments, as well as in non-medical environments, with the most popular being the Neurosky^®^ and Emotiv^®^, which generate measurements of the ratios of the frequency bands of brain waves. BCIs or BMIs, by definition, are interfaces that use brain signals to control close-loop systems in real time. The variability of the inter- and intrapersonal EEG signal has generated, over several decades, discomfort for scientific purposes. Only in the last decade, with the advancement of technology and the advent of low-cost portable EEG headsets, has it been possible to popularize the capture of the EEG signal in various non-laboratory contexts and generate comparative patterns between experiments from pre-defined levels and scales by these equipments.

The largest global technology companies, Facebook and Tesla, have already shown recent interest in research with neural signals. Facebook wants to provide disabled people’s ability to communicate by capturing cortical neural signals in high spatial resolution to read their thoughts, decoding perceived and produced speech of up to 100 words per minute in real time (Moses et al., 2019). Tesla and SpaceX intend to generate superfine structures, which make it possible to connect thousands of flexible electrodes placed in the brain in arrays and threads, better recognizing neural activity with a spatial resolution much higher than that of the EEG (Musk, 2019).

A recent survey draw attention to the complexity of integrating the elements, as well as pointing to the future direction of research with neural signals. The work by the company Neurable is noteworthy, featuring a game controlled solely by a neural signal, captured via the company’s own headset, which combines HMD with Virtual reality (HMD-VR) and EEG headset in a single device. The company has received a recent multi-million dollar investment, and intends to take the lead in producing EEG headsets for everyday use in the context of the Internet of Things (IoT) (The Lancet, 2019). EEG headsets normally work with one to eight electrodes on the scalp surface to record EEG signal, however, the 10–20 standardization, so named for distancing 10 or 20% of the longitudinal and latitudinal measurements from the head surface. In systems with higher resolution, the modified combinatorial nomenclature (MCN) originates, filling spaces between the traditional electrode arrangement by the 10–10 pattern, with 64 electrodes or a little more, covering brain areas: F – Frontal and combinations (Fp, Fc, AF, FT), P – Parietal (also PO), T – Temporal (also TP), C – Central (also CP), O – Occipital.

The next subsection, we attempt to cover the main methods proposed in literature for artifact removal, feature extraction and classification on EEG. We firstly review the characteristic of EEG signal and the types of artifacts existed. Then, we present the widely applied techniques, their advantage and drawbacks regarding the characteristic of EEG signal. We believe that the knowledge provided in this review can help to determine an computational technique, which satisfies the necessary requirements for a particular application.

#### Electrophysiological-Based Analytical Methods

The main detections on the EEG signal are related to changes in the natural neural signal due to external stimuli, these changes are detected by patterns of signal alteration, called paradigms. The main EEG signal detection paradigms are event-related potentials (ERPs), Sensory Evoked Potentials (SEPs) and synchronization or desynchronization event-related potentials (ERS/ERD). The extraction of these rhythms, and special potentials related to events, are of great importance in the research and application of the brain signal detected via EEG. However, several artifacts are found in this signal, such as the electrocardiographic (ECG), electrooculographic (EOG), and electromyographic (EMG) signals, common interference, baseline drift and other various random noises. In the analysis of these non-stationary, non-linear and stochastic electrophysiological signals, it is necessary to correctly manipulate the EEG signal in the removal of artifacts (to have a good signal-to-noise ratio – SNR), extraction of characteristics and classification of the neural signals obtained through the application of analytical methods.

The feature extraction from the recorded EEG is challenging, as subjective experimental and individual characteristics can influence the source artifacts, contaminating the EEG signal and making it difficult to accurately extract brain waves. First, a visual inspection to remove artifacts must be done, which requires the operator’s visual acuity. Several forms of automatic detection of artifacts are explored (examples are shown in Supplementary Table 1), in order to reduce the subjectivity of the operator in the detection of artifacts, such as Independent Component Analysis (ICA), fast independent component analysis (FastICA), regression analysis (RA), adaptive filters (AF), machine learning techniques – such as autoregressive models – and feature scaling, such as discrete waveform transform (DWT). However, they are methods and models that, for the most part, require great computational cost and are not applicable to EEG headsets or devices with one channel. The downsampling alternative is one of the first strategies applied in this context.

The computational analysis of the EEG signal cannot be detached from any step of signal processing, even if this requires great computational effort. Recent computational tools of analytical methods are related to spectral (frequency) analysis. The frequency bands of the EEG signal (α, β, δ, θ, γ) are distinct and analyzed by statistical tools, in relation to their spectrum amplitude, coherence, latency, etc. Automatic classification (recognition) of EEG is the inevitable direction in analyzing these signals, however, quality criteria of the signal and its disturbances remain not categorically analyzed by neuroscientists in their research (Furman et al., 2019), as the superposition of elementary sources of pyramidal neurons. These elements may be the reason for recommending individual models (or user-dependents) so that a generalization is challenging in the current context. It points out that classifiers are trained at the group level with performance similar to the individual training level (Wang D. et al., 2011). Cross person classifiers were satisfactory in classifying non-linear features in EEG signals in the work of Stikic et al. (2014) and five other works reported in Supplementary Table 1.

Although we have not yet reached the ultimate stage regarding the EEG signal processing, several recent alternatives are explored in the feature extraction. Statistical analysis has gained great space in scientific research, spectral analysis – raw/pre-processed signals, discrete wavelet transform (DWT), wavelet packet decomposition (WPD). These decomposition techniques, DWT and WPD, are efficacious because significant information is carried in different EEG bands (Kevric and Subasi, 2017). Our study found results in accordance with the literature (Khosla et al., 2020), in relation to the most used tools for feature extraction, for a similar sample of articles (44).

For the classification stage, some most common techniques are cited in Supplementary Table 1, such as Support Vector Machine (SVM), Principal Component Analysis (PCA), Common Spatial Pattern (CSP), Convolutional Neural Network (CNN), Linear Discriminant Analysis (LDA), K-means clustering, Naïve Bayes (NB), Mismatch Negativity (MMN) – and statistical tests – ANOVA, Student *t*-test. The MMN is a specific ERP component, which is observed at fronto-central sites as a combination of the 2 different preattentive components: MMN with P3a and the negative-going MMN component (peak at approximately 150–250 ms from the onset of the deviant stimuli in a passive auditory oddball paradigm) followed by the positive-going P3a component (peak at approximately 220–280 ms). This behavior reflects a subsequent attention-orienting process in the neurophysiological performance of MMN to the P3a (Chung et al., 2018). Sequential forward floating selection/search (SFFS) is a procedure applied in classification analysis, which dynamically changes the number of selected features, evaluating the best performance in the process. This procedure shows up in two studies.

Some particular cases are less common. In one study, for emotion recognition using an EEG signal, the fractal dimension of raw signals has been implemented to extract the feature using the Higuchi technique (Kaur et al., 2018). As classifiers, linear embedding (LLE), support vector clustering (SVC) and support vector data description (SVDD) are proposed in one work as a technique to find the low-dimensional manifold in the high-dimensional EEG feature space in order to extract the representative EEG markers from different cortical regions (Yin and Zhang, 2014). The Multi Layer Perceptron (MLP) is described in one study (Lamti et al., 2016), it is a class in artificial neural network (ANN) and has 4 neurons output corresponding to the number of emotions. The SVM technique applied with radial basis function (RBF) kernel shows up in one study. For paradigm analysis, there are in Supplementary Table 1 the possibilities for EEG features: Steady State Visually Evoked Potential (SSVEP) and Sensory Evoked Potential – SEP – (N80, N200, P200, P50, P300, P600) are the most common.

#### Immersion Systems and User Experience

Regarding the changes in EEG results in the concentration and immersion states (Ray and Cole, 1985) suggested that the concentration state is associated with Alpha waves Immersion definitions. First, the definition of immersion must be considered. Immersion comprises relating engagement in the user experience, taking into account the environment in which it is inserted. Immersion can be a complex concept, with multiple definitions, encompassing notions of usability (Baek et al., 2019), emotional response (Manetta and Blade, 1995), quality of experience (QoE) (Choy et al., 2021), fun in games (Jennett et al., 2008), flow (Csikszentmihalyi, 1991), among others. These last authors, Jennett et al. (2008), indicate that immersion should contain the following characteristics: lack of awareness of time, loss of awareness of the real world, involvement and sense of being in the task environment. In this sense, it is not enough to have selective/oriented attention to the virtual environment, but levels of immersion can be denoted. The author Jennett points out five components of immersion, in the context of games: control, challenge, real world dissociation, emotional involvement and coming cognitive involvement. Regarding these components, we can work with the concept of virtual reality (VR), which requires immersion and interaction with the virtual environment, but not necessarily all VR devices will require the same level of immersion (Robertson et al., 2002). It is a common sense that more immersive is the experience, more attention allocation is dispended.

Head-mounted-display devices (HMD) based on VR systems can make the user achieve the greatest degree of immersion, offering a great sense of being present, thus increasing the allocation of attention demand. The HMD is a VR headset that positions screens in front of the user’s eyes, closing the sides and hanging over the individual’s head, making it impossible to see outside the virtual environment projected on the screens. These headsets have built-in gyroscopes, causing the user’s head movement to position the virtual environment according to the user’s direction. This technology incorporates Stereoscopic 3D (S3D), which has seen great recent growth, as in the Facebook Oculus (Egliston and Carter, 2020), Oculus Rift and HTC VIVE (Borrego et al., 2018) equipment. Despite enabling great user immersion, it can also generate great visual fatigue and visually induced motion sickness (VIMS) (Choy et al., 2021).

Virtual reality devices (VRs), can be classified into desktop-based VR systems, fully immersed VR systems, and distributed VR systems (Liu et al., 2017). These differences are necessary in the classification of immersive VR environments, since the user, when watching a 2D or 3D video, without interaction with the virtual environment by any control, is not facing an immersive environment – non-immersive VR, or desktop- based VR systems. Semi immersive VR devices, or distributed VR systems, allow a mixed view of the virtual environment, as with the use of 3D glasses. In the fully immersed VR system, the immersion device must be an HMD or be placed in a cave automatic virtual environment (CAVE), which is a room covered by screens on all walls (Li et al., 2020b). In order to understand the context of immersive environments (and not just games), the three levels of immersion are identified: 1 – engagement – initial level of immersion, making the user invest time, effort and attention, but no further involvement is noticed; 2 – engrossment – second level of immersion, in which the user gets attached with an emotionally immersive environment, and makes that experience an important part of their attention, effort and time and 3 – total immersion – total sense of being present (SoP), the highest level of immersion, with the user’s complete immersion, making him feel inside the environment and the possibility of flow.

Analogous to immersion, concentration brings the individual into a very similar state of mind, which requires directing attention toward a particular task. However, the authors Lim, Yeo and Yoon (Lim et al., 2019) conducted a study in an attempt to differentiate the states of concentration and immersion through the EEG signal. In concentration, subjects should focus on a red dot in the center of the screen, while in immersion they should focus on playing a VR game. The beta waves rose in both the concentration and immersion states in the frontal and occipital lobes, increasing further in immersion. The alpha waves showed only decay in both concentration and immersion states, at rest times between tasks. Factors that stimulate the immersion process or greater attention of the individual in the required activity were not confronted in the study, or factors that call the individual’s visual attention, causing saccade, were not investigated, in order to assess the individual’s distraction during recording of the EEG signals.

One factor that stimulates the individual’s immersion process in the virtual environment is the individual’s presence in the virtual environment. This is possible through an avatar, which is defined as a direct extension of ourselves as they are a close resemblance of what we experience in the real world (Waltemate et al., 2018). When transposing to the virtual world, identifying itself as an avatar, we are facing an *illusion of virtual body ownership* (IVBO) (Slater et al., 2008). Even temporarily, the individual with the help of the avatar may present new behavior or have a self-image different from what they actually perceive, called the *Proteus* effect (Yee and Bailenson, 2007). When measuring the impact of the IVBO relationship and the immersion levels of the environments, authors found that environments with full immersion are strongly recommended in the emotional response process, increasing the SoP (Waltemate et al., 2018). Games are the main way to explore the individual’s presence in the virtual environment. More focused on medical attention, exergames (VR-based exercises) have been a recent investigation into the approach of VR technologies, scientific investigation of physiological signals and therapies, often with the use of avatars.

Exergames have been a recent outlet for therapy for people with a psychological disorder or educational tool. The use of exergames in treatments has been well accepted for being affordable in terms of time, costs and a sense of achievement when achieving the proposed goals. In non-patient individuals, it is especially interesting in order to gain a greater understanding of the effects of greater engagement in long (sustained-attention situation) and repetitive games (Wouters et al., 2013). The EEG is used in the context of exergames, in the neurofeedback process, as it awakens the individual’s ability to learn important steps in the game, being informed of the cognitive state of interest. When there is a desired action, the individual is rewarded as well as being punished for unwanted behavior in the game. This feedback can be visual or auditory, but, as mentioned earlier, the visual stimulus is more recurrent. The neurofeedback technique stimulates engagement and, consequently, attention/arousal in the individual.

However, the correct application of EEG signal-based technologies in the context of exergames, or perception of 3D content, with validation and effectiveness is currently not completely clear. There is, therefore, a barrier between understanding the results extracted from the EEG signal and applying exergames or virtual games in general (Coenen et al., 2020). In general, there are proposals for the perception of 3D content using a widescreen and HMD to achieve better visual attention (Clemente et al., 2014; Choy et al., 2021). However, standardizations of the design requirements of subjective methodologies in this sense remain uncertain, so that scientific results are better explored and have better quality in their conduction.

## Methods

### Study Design

The breakdown of the research carried out in this study is intended to restrict the scientific information found in excellent journals, without, however, pointing out the origins of the work. The aim is to discover whether many reference works in the literature encompass all the descriptors pointed out in the research strategy section of the articles and how they relate these characteristics. The possibilities of relating the collected signals, the extraction of characteristics and the paradigm/external stimulus used are diverse. In this work, we propose to update on the most assertive investigations that relate attention-based elements, with immersive environments and technologies that capture the neural signal in an easier way (EEG signal), showing the main paths that need to be better explored.

The studies were processed according to: study sample; duration of visual stimulus; device for capturing the EEG signal with number of channels; signal sampling frequency; EEG device wired or wireless communication; location of acquisition electrodes; processing technique used to extract characteristics of the EEG signal; which is the main investigation associated with the EEG signal (attention allocation, workload, drive simulation, fatigue, game, VR variables, serious games); level of immersion in virtual environments; presence of visual/sound stimulus to constitute the extraction of EEG signal characteristics, type of data processing (online or offline), whether data processing is user-dependent or not, in addition to the main results found.

### Data Sources and Studies Selection

The platforms for scientific articles consulted in October 2020 were: PubMed, MEDLINE, Scopus, Web of Science, IEEE Xplore, and ScienceDirect. The search was sorted by a combination of terms: Attention, Fatigue, Workload, Immersion, Game, Virtual Reality, Augmented Reality, Mixed Reality, Extended Reality, EEG, BCI, Brain Computer Interface, Electroencephalography. No suitable MeSH (Medical Subject Headings) terms have been identified. ‘On the subject’ filter were used, in order to prevent finding articles citing the terms of interest in another context. Inclusion criteria were: only peer-reviewed journal articles written in English; use of surface EEG to access information on the variation of attention/workload/fatigue in the use of immersive systems; use of auditory or visual paradigms in interacting with the system; motor or neural control of the virtual interface.

A total of 85 articles were identified between 1986 and 2020, with only 76 being peer-reviewed. In order to generate a fair comparison between different studies and minimize bias in the interpretation of results, in this study, the discussion will be centered on experiments with EEG signal collection in non-clinical adult populations, in response to generated visual and auditory stimuli, when interacting/controlling virtual systems, with some degree of immersion in the virtual system. Purely review articles were excluded, one article written only in Chinese was excluded, as well as articles with incremental contribution by the same authors.

**FIGURE 2:**
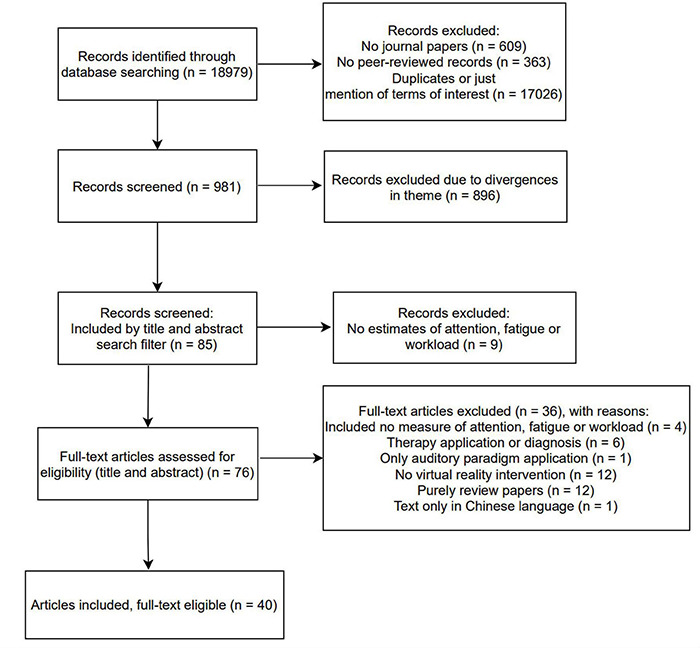
PRISMA flow chart scoping attention allocation, virtual reality, cognitive workload and fatigue.

As we are interested in evaluating articles that deal with the investigation of attention, workload, fatigue and their derivatives in the manipulation of immersion systems, were also excluded: articles that only include measurements of signals other than EEG, articles using only sound paradigms, articles with actual exposures to activities (not simulated ones), articles with virtual reality interventions in the elderly and articles studying the effect on therapy, or diagnosis, of diseases such as: dementia, Attention Deficit Hyperactivity Disorder – ADHD, mild cognitive impairment, autism, stroke. As a result of the selection by the PRISMA method, see Figure 2, we identified 40 articles, the oldest from the year 2011 and the most recent from the year 2020, dealing with immersion systems and investigation through the EEG of attention variation attributes, cognitive load or user fatigue. Next, we will present the main research topics extracted from these works, we will summarize the scientific findings and point out future directions in the subject.

## Immersion Environments Measures on Attention, Workload and Fatigue Based on Electrophysiological Signal Analysis – Results

In line with the work of Brouwer et al. (2015), we adopted the six recommendations to avoid common pitfalls in the use of electrophysiological signals that reflect cognitive or affective state. In the database selected in this study, we investigated some analyses: Work analysis considering the test protocols phases (stimulus – duration and subjects); work analysis considering the EEG recording phase (equipment – frequency, location of electrodes); Work analysis considering the Filtering phase of artifacts; Work analysis considering the feature extraction phase; Work analysis considering the Classification phase (online or offline methods – or both –, user-dependent or independent outcomes and analytical analysis method of the EEG signal). In order to standardize the findings of the scientific works analyzed, we built Supplementary Table 1, which list the works in order of year of publication, pointing out the analyzed characteristics of each one. The classification of articles according to the analyzes used is explained below.

### Classification

#### User-Dependent/Independent

The data classification process can be done with models adhered to the analyzed subjects, in an individual or generalized way. When the model is individualized, generally better results are presented, but there is a loss in processing generalization, which can affect the comparison of results among individuals. In Supplementary Table 1 we detail which analyzed works in this study relate their models and samples in an objective or subjective way.

#### Main Investigation Through the Electrophysiological Signal

The investigations analyzed in the articles were divided into the following categories: attention allocation (AA) (which may be accompanied by the terms ‘education,’ ‘game’ and ‘internal and external,’ depending on the context of the article); VR Variables (VRV); Drive simulation; Learning; Workload; Fatigue; and some combinations of these terms, depending on the main investigations of interest to the scientific work. These terminologies were annotated in this section according to our perception of the main investigation of the addressed article. What is its main focus of analysis and main output variables presented.

The columns *Stimulus, Subjects, Device and N. of channels, sample frequency and electrodes location* refer to the characteristics of the EEG study device, describing how many times and the duration of the visual stimulus exposed to the sample, in order to generate the EEG signals recorded in the study. Which EEG device was used in data recording, as well as how many channels, sampling frequency of signal recording were also mentioned. The placement of the electrodes is also evidenced in the aforementioned column, in order to facilitate the comparison among works, for the investigated brain lobes. The *Communication* column differentiates the studies between those with wireless (NW) and wired (W) communication. This communication refers to the EEG device and how the recorded data is sent to the destination computer.

#### Immersion Level

In this column, we bring together the definitions discussed in section D. We adopted as ‘Engagement’ the works in which there is little interaction with the individual, or the virtual environment is non-immersive and research was conducted with desktop-based VR systems, without a virtual scenario close to real one or the graphics did not show robustness. Games without avatars or tetris-like are included in this context. ‘Engrossment’ included works with more robust graphics, conducted in semi-immersive environments, with the presence of an avatar or not, in which individuals had a greater involvement with the virtual scenario, but it was not yet a condition of total immersion. In the ‘Total Immersion’ classification, the individual was exposed to well-refined graphics, with scenarios close to reality, in the presence of avatars, conducted by HMD or inserted in CAVEs.

#### Electrophysiological-Based Artifact Filtering, Electrophysiological Features, Feature Extraction and Analytical Methods

Several analytical methods can be applied in the treatment of EEG signals. Section *C.(i).* lists all the analytical methods, artifact filtering techniques and feature extraction found in the analyzed works, mentioned here for the purpose of characterize and allow comparison of related articles.

#### Offline/Online Signal Processing System – OFF vs. ON Column

Offline signal processing (OFF) describe systems that perform the complete EEG signal processing after the signal recording and completion of the data collection phase. Online processing (ON), on the other hand, is plausible in neurofeedback, for example, where results are found in real time, due to the EEG signal processing in real time up to the user to respond to the activity in the moment of performance.

Supplementary Table 1 summarizes the 40 analyzed works addressing a number of methodological settings including: experimental protocol (subjects, stimulus duration and repetitions number), experiment immersion level and EEG recording data (equipment, sampling frequency, electrodes location and mean of communication to send data to the computer). The Artifact Filtering stage is highlighted (computational techniques used to de-noise, select or pre-processing the EEG signal of interest), considering the EEG Features (features extracted from EEG signal), the Features Extraction (computational techniques to extract EEG features of interest), the Analytical Method/Classifier of the EEG signal stage (analytical/computational techniques applied to classify the EEG signal events designated in EEG features), and considering the study methods of recording and processing data (online – ON – or offline – OFF – methods – or both –, user-dependent – UD – user-independent – UIN – or both outcomes). These detailed information addressing each one of the 40 analyzed works is done in order to map the main methodology settings and punctuate what to compare in them in this context.

The table below is divided into immersion levels due to the methodological works characteristics and the experimental conditions exposed to the individuals (passive games, non-avatar interface, HMD, CAVE, etc.). The level of total immersion will not necessarily generate higher levels of attention allocation as a result than in engagement or engrossment immersion levels. The presentation of this content division is intended to provide to the reader tools of which analytical strategies and tools were used to identify the attention allocation and which can be reproduced by him for comparison purposes. Next, we discuss the methodologies used in the analyzed studies.

### Main Findings

Below we summarize the main findings of this study aiming to converge to a set of methodological parameters commonly used to attention detection, workload and fatigue approaches from EEG signal analysis. In the Supplementary Table 1, we can observe that half of the studies (20) developed their methodologies with low immersion in the virtual environment, only at the level of engagement. The stimulus duration and times of stimulus on the experiments vary a lot among methodologies, as well as the results are quite different from each other.

At the engagement level of immersion, the main investigations of the EEG signal are associated with the attention allocation (6) and the evaluation of variables of the virtual reality system (VRVs) (6), one study concurrently analyzed fatigue and attention allocation variables and two studies associated an investigation of learning and workload. As other investigations for this level of immersion are in relation to the study of behavior in driving simulation environments (5), assessment of the workload level (2) and, finally, fatigue was the main investigation alone in only one (1) work. In the second level of immersion considered in this work, engrossment, seven (7) studies investigated variables on: attention allocation (2), workload (1) and evaluation of the virtual reality system (4). At the level of complete immersion, total immersion, the allocation of attention is the main investigation in six (6) works, and it is a concomitant investigation with workload in the situation of drive simulation (1) and VRV (2). VRV is the main investigation in one (1) work purely, and concomitant to workload (1). Workload is the primary investigation into only one (1) work or concomitant fatigue (1).

The main findings from the methodologies adopted in the works are pointed out below.

• Studies dealing with VRV analyzed two gaming conditions for the same sample at *engagement level*:
  ◯ for different levels of a game, no significant differences were found in α and β waves for the subjects’ brainwave power data (Coenen et al., 2020), while (Cowley and Ravaja, 2014) identified decreased delta power and relatively balanced fronto-hemispheric alpha power in the five levels analyzed;
  ◯ for the same game and different control manners, it was identified that the visual evoked potential (SSVEP) at the frequency of 12Hz was satisfactory for controlling the interface, noting the fatigue of individuals in this type of control based on questionnaires (Leite et al., 2018), while (Asensio-Cubero et al., 2016) compared classification methods for controlling a BCI through body movements, in a multiresolution analysis;
  ◯ when comparing a virtual simulation and a real game, significant lower power data were found for the θ wave in the condition of the simulated scenario (Fuentes-García et al., 2019); for different cognitive load demands in the game, as learning increases, the best predictors were frontal alpha power and alpha and delta ERSPs, but not P300 (Mathewson et al., 2012); the authors (X. Yang et al., 2018) propose the comparison of immersive VR conditions and paper-pencil schemes. Based on EEG variables, the results showed that participants in the immersive VR condition maintained a more stable focus or attention than others without VR immersion.
  ◯ the sense of being present was evaluated at *engrossment level*: identified by more pronounced N200 and P300 potentials in the fronto-central and occipital electrodes, were features regarding the exposed virtual environment, but not modulated by the different tasks requested (Vogt et al., 2015), as for the two screen types analyzed (Clemente et al., 2014), the common desktop screen and the high-resolution power wall screen, significant differences were found in θ and α increase in navigation conditions (screen with greater immersion). Still on active or passive user action identification (Chen et al., 2014) investigate the P300 and its differentiation in these two states, using dynamic causal modeling, which suggests that passive and active P300 share the same parietal-frontal neural network for attentional control.
  ◯ In order to compare neurofeedback systems in 2D or 3D games (Wang Q. et al., 2011) propose outputs with fractal dimensions in order to estimate concentration levels of individuals in these environments. The authors (Li et al., 2020b), monitored the usability of VR systems in 2D and 3D situations, resulting in greater allocation of attention to the 3D scenery. Berger and Davelaar (2018) investigated the exposure of two groups to 2D and 3D environments separately, in order to confront, by means of α, whether exposure to learning tasks would indicate cortical inactivity or cortical processing efficiency, the latter being the final association with efficient neurocognitive processing in the 3D environment.
• In attention allocation findings, most at *engagement level*:
  ◯ in the application of action games to two distinct samples of players and non-players, aspects of selective attention were investigated, as a function of inhibitory control, in which α, β, and γ waves performed better in the sample of players than non-players (Hazarika et al., 2018);
  ◯ a significant improvement in attention was also found through the θ/β bands power ratio (Yang et al., 2018);
  ◯ increased cortical activation (Lee et al., 2015);
  ◯ identification of P300 in learning contexts (Rohani and Puthusserypady, 2015) and
  ◯ multivariate analysis (Myrden and Chau, 2015) com with ongoing user experience.
  ◯ the states of concentration and immersion were compared at *engrossment level*, in the concentration task, a specific point on the screen was required for focus, while in the immersion task the task was to control the entire game. Significant differences in α, θ, and β were identified for the different tasks (Lim et al., 2019);
  ◯ In HMD systems investigations, at *total immersion* level: to assess the theory of attention restoration (ART) in two different groups, given the variable response time and EEG, θ and parietal P3b metrics (Li et al., 2020d) and when faced with passive auditory stimuli, in the assessment of mismatch negativity (MMN)/P3a (Chung et al., 2018), pointing to highly restorative experience; in six different virtual environments (Gao et al., 2019), identifying improvements in the state of restoration and fatigue relief; (Peterson et al., 2018) analyzed individuals during exposure or not to VR by HMD during beam-walking, as a result, through peaks of EEG amplitude, they found that VR exposure may increase physiological stress during dynamic balance tasks and may impair physical and cognitive performance during balance; in addition to the investigation of feature-based attention (FBA) through SSVEPs (Chu and D’Zmura, 2019), pointing out that these potentials are strongly influenced by flicker stimulus in the peripheral visual field.
• Driving simulation findings approaches can help prevent accidents due to human fails are, in some conditions, most at *engagement level*:
  ◯ supervised and unsupervised training strategies, pointing out that combinations of classifiers in specific frequency bands can identify situations of mental load change, such as increase in the frontal region of θ (Yin and Zhang, 2014);
  ◯ evaluating the high mental load in the face of congruent and non-congruent stimuli in a joint task while driving, the identification of P300/P600 was identified as a result in the presence of difficulty in orienting attention in multitasking conditions (Causse et al., 2016);
  ◯ the significant increase in α and θ, as well as the significant decrease in β are indicative of fatigue during long periods of monotonous driving simulation (Jagannath and Balasubramanian, 2014);
  ◯ from brain spectral components, in which high accuracy was identified regarding the focus of attention (Wang Y. K. et al., 2015) and fluctuations in behavior (Touryan et al., 2016), in order to enable systems to dynamically assess the attention spent during the driving simulation.
  ◯ flight training, in *total immersion* level, challenges are proposed at three levels in the study by Jaquess et al. (2017), through the amplitude of potentials N1, P2 and P3 (attention allocation) and measures of cortical activation (workload), empirically corroborated the notion that cognitive workload and attentional reserve are inversely related.
• In investigations purely of workload and fatigue, modulations in the EEG signal were distinguished in the investigations:
  ◯ at four different levels of mental demand, the attenuation of P300 was greater as there was greater workload demand (Yu et al., 2015);
  ◯ analyzing two workload scenarios, there was a significant increase in the frontal region and a significant decrease in the occipital region of activity θ for the scenario of higher cognitive load (Savage et al., 2013).
  ◯ For the investigation of fatigue, the association of different classifiers to identify the P300 correlated with different levels of fatigue can be useful to monitor virtual wheelchair control systems, as shown in Lamti et al. (2016) study.
• Inside CAVE simulations:
  ◯ Magosso et al. (2019) applied two flight cabin simulations. In both experiments, participants were exposed to tasks with mental demand at different levels. The authors, through the processing of the EEG signal, identified that α decreases when there is an external visual stimulus and when there is a very high arithmetic demand. On the other hand, α greatly increases during the purely mental task in VR immersion is noted.
  ◯ Pereira et al. (2018) analyzed the Olympic shooting scenario, the participants performed the same experiment and, at the end, they identified that novice shooters with lower pre-shooting α have better performance competition in a VR scenario.

**FIGURE 3:**
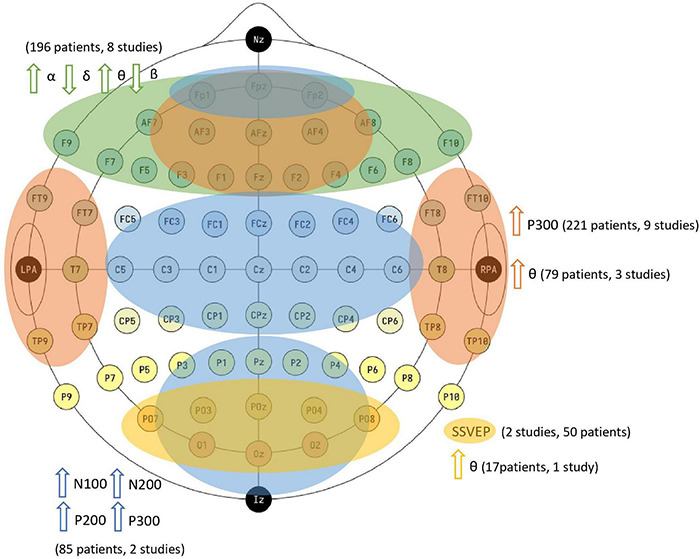
Main predictors for identification of attention allocation, higher immersion states and higher cognitive load effects as found in the database. Image of public domain, by Laurens R. Krol. EEG electrode positions in the 10–10 system using modified combinatorial nomenclature. The electrode sites are color-coded according to the lobes of the brain which their labels (F, C, P, O, and T) represent. The head indicates the location of the fiducials: the nasion (Nz), the (left) pre-auricular point (LPA), the (right) pre-auricular point (RPA), and the inion (Iz). About the studies cited, up arrows are for increase of those features and down arrows for decrease. The studies and patients are detach from Supplementary Table 1, here are grouped in methodologies and main predictors to indicated attention allocation and similar investigated states. The green color shades the frontal and pre-frontal areas (electrodes: Fp1, Fp2, Fpz, F1–F10, AF3, AF4, AF7, AF8, AFz, and Fz), in these areas the eight studies described in the ‘Main Findings’ section identify rising α and θ and declining δ and β in recognized attention allocation states; orange color shade is in fronto-parietal areas (Fp1, Fp2, Fpz, F1–F10, AF3, AF4, AF7, AF8, AFz, Fz, T7, T8, FT7–FT10, and TP7–TP10), in these areas eleven studies identify attention allocation by means of P300 presence or θ rising; blue color shade is in fronto-central-occipital (Fp1, Fp2, Fpz, FCz, C1–C6, FC1–FC6, CP1–CP6, Cz, CPz, O1, O2, PO3, PO4, PO7, and PO8), in these areas two studies identify from presence of event-related potentials N100, N200, P200, and P300 assigning attention allocation in the proposed methodologies; and yellow color shade comprises occipital area (O1, O2, PO3, PO4, PO7, and PO8), in three studies this area is related to the attention allocation investigation, by means of SSVEP technique or θ rising. The electrodes associated with those regions are processed by means of EEG signals, identifying to record changes in frequency bands and ERPs associated with changes in the attention allocation.

In order to summarize the main findings mentioned above, Figure 3 below illustrates the regions of the main predictors of identification of attention allocation, situations of more immersion states and effects of greater cognitive load. The indication of increased θ/β bands power ratio is supported by these findings in frontal, parietal and occipital areas, according to the works pointed out above and highlighted in Figure 3.

## Discussion and Conclusion

Although 20% of the works analyzed in this study work with machine intelligence classifiers to extract information from the mechanism of attention, recent studies have shown that powerful robust outcomes are extracted when creating models to encode and decode attention in Artificial Neural Networks (ANNs) (Cho et al., 2015). However, it is important not to overestimate the results achieved with machine intelligence, nor to extrapolate to a single, universal model. The current state of the art has not yet reached this level (Li et al., 2021). Wang Y. et al. (2020) also points out that the way forward with machine learning and models for detecting attention and fatigue are better when individualized, especially for risky situations. Likewise, it is not interesting to underestimate models that do not use machine learning, since they are less complex, have a lower computational cost and often interfere less in the source signal, causing less bias in the obtained results. By bringing neuroscientists together, observing opportunities and considering the possible pitfalls in each step of analyzing the variables measured by the EEG, developers can reach new goals of making everyday tools based on the neural signal more common, through IoT technologies and wearable devices.

Head-mounted-display devices with virtual reality environments extend SoP and attention, leaving behind 2D applications without immersion and user interaction (Li et al., 2020b). A recent work (Wan et al., 2021) also defends that, through EEG signal outputs for detecting working memory and attention state, applications involving VR environments are greater to those with 3D interaction, having a better impact on the user’s cognitive ability. It is important to consider these characteristics in modern devices, regulating cognitive load and visual fatigue, in view of greater engagement and better access to sustained-attention in new generations. Even in VR scenarios with little interaction, a profound effect is identified in the alpha band as indicative of the individual’s isolation from the external environment, promoting attentional orientation to his internal aspects. The authors Magosso et al. (2019) corroborate these perspectives, showing that this occurs in the need for greater mental effort. Several developments in psychophysiological investigations can be studied based on the results presented in the cited works. There is still, however, a lack of high-resolution EEG approaches, presenting connectivity of cortical regions of interest to access information on immersion, attention, cognitive load and fatigue to concurrent stimuli, as the neural circuits or processes of the attention should not be studied in isolation (Amso and Scerif, 2015). For example, since workload classification algorithms often utilize the ratio of power in the clinical frequency bands, the modeling approach designed to identify level of alertness from EEG spectra would also capture some aspect of mental workload.

In this review, we present the analysis of works dated from 2011 to 2020, which proposed to process EEG signals in the context of immersion systems, analyzing the cognitive load or attention expended in the interaction with such systems. Our analysis was designed from the perspectives of (1) identifying the steps of analysis and processing of EEG signals in relation to virtual interaction environments and (2) pointing out the main extracted characteristics and tools associated with the immersion system using the signal EEG. EEG-based signal analysis are indicated to obtain objective results and accurate measures of fatigue (mental and visual), cognitive workload and attentional orientation. As presented in this review, several terminologies are associated with the study of attention, some involving attention cue and others exploring the origin of the external or internal stimulus that caused the elevation of brain waves as a result of the attention allocation. The perception of attention in the EEG signal will depend more on the region analyzed (i.e., on the placement of the electrodes) and less on the analytical method used in the extraction of signal characteristics. Regarding the results presented in Supplementary Table 1, this can be observed, since different methodologies are applied and different outputs analyzed, not complying with the input variables, analysis and outputs coordinated among the analyzed works. Thus, it is possible to observe a large amount in the variation of the features analyzed with loss of information in the different processes interposed in the different methodologies. As a result of this diversity, the behavior estimate remains a black box in the methodological perception.

The EEG signal is maximally correlated with measured behavior, regardless of the perceptual, cognitive, motor or artifact process from which they are generated. Thus, there is no clear method for predicting how well a specific model will translate into a new task. In Supplementary Table 1, we specify the times of EEG signals recorded, considering the application of one or more tasks for a short or long period of time. With this information, we can outline the studies and their findings counting long period of task time associated with the EEG signal that can generate information in order to build a hierarchy of neural characteristics to maximize the universality of EEG-based performance prediction models. While the intermediate constructs of workload and engagement can be helpful, the stability of constituent neural characteristics, especially under stress and fatigue, remains unclear under long period of time applications.

As future direction, we can list the main recommended aspects that surround and must follow attention detection/investigation through EEG-signal:

i. create/use a recognized great data-driven model, user-independent;
ii. analyze constructs separately, avoiding controlled environment conditions;
iii. search for new EEG-signal devices, they can be very specific for detecting attention in some applications;
iv. Avoid new terminologies, focus on using what already exists to explain new strategies/methodologies;
v. Avoid overprocessing EEG signal, avoiding inputting new bias;
vi. Improve actual free-viewing approaches;
vii. Vary methodologies to study attention allocation in different immersion levels and different cognitive load conditions. In this way you will obtain more results of attention allocation in brain signals out of different triggers;
viii. Avoid gender bias, besides only 10 out of 40 studies showed in Supplementary Table 1 have balanced male/female proportion. Male gender prevails in most studies;
ix. Investigate attention by means of ERP components to identify attention allocation through N100, N200, P100 and P300 in fronto-central-occipital brain areas (Vogt et al., 2015; Jaquess et al., 2017); P300 in fronto-parietal brain areas (Rohani and Puthusserypady, 2015; Vogt et al., 2015; Yu et al., 2015; Causse et al., 2016; Chen et al., 2016; Lamti et al., 2016; Jaquess et al., 2017; Chung et al., 2018; Li et al., 2020d) and SSVEP component in occipital area (Leite et al., 2018; Chu and D’Zmura, 2019) as we cited in Figure 3;
x. Investigate attention by means of brain waves increase, decrease and beta (12–31.25 Hz)/theta (3–8 Hz) ratio in fronto-parietal brain areas, θ (Clemente et al., 2014; Fuentes-García et al., 2019; Lim et al., 2019), in occipital area, θ (Savage et al., 2013), in frontal area, αβθδ (Clemente et al., 2014; Cowley and Ravaja, 2014; Jagannath and Balasubramanian, 2014; Yin and Zhang, 2014; Lee et al., 2015; Hazarika et al., 2018; Lim et al., 2019; Li et al., 2020d) as we cited in Figure 3.

Explaining better this list, we indicate that data-driven models of behavior could operationally defined constructs to better understand what constructs are incorporated in a predictive model of behavior and their limitation (what processes are fundamentally task-specific). In this case, a more detailed analysis of the behavior, additional EEG processing, and the inclusion of other physiological measures are needed. For example, some aspects of the observed behavior may be more strongly associated with one construct (e.g., fatigue or stress) whereas other aspects are more directly linked to a collection of task-specific neural processes. New technologies may benefit from identifying common neural inseparable processes in some cognitive constructs to explain behavior, such as brain-computer interfaces (BCIs), which have been a field of great interest in science, emerging as commercial products. New commercial approaches to BCIs will be seen in the market, as large technology investment programs, such as Horizon 2020, have concentrated many efforts and investments in this field.

As we have presented, there is a lack of standardization in the terminologies dealt with in neuroscience, evaluating engagement and EEG-based interfaces. There is also a trap when processing the EEG signal under controlled conditions, while it must be inserted in everyday applications, in contact with natural stimuli from its environment, in the context of free-viewing. Concerning this controlled conditions, HMD-VR has two inherent advantages with respect to enhancing one’s neural state that each deal with unique sources of irrelevant information: the ability to effectively limit influences of external distraction on attention and the ability to heighten engagement internally to remediate internal distraction. This path will be the greatest potential use of EEG-based device applications, in detecting the individual’s attention and engagement, in everyday, natural and virtual use.

## Author Contributions

RS and EN contributed to conception and design of the study. RS organized the database, performed the analysis, wrote the first draft of the manuscript and sections of the manuscript. All authors contributed to manuscript revision, read, and approved the submitted version.

## Conflict of Interest

The authors declare that the research was conducted in the absence of any commercial or financial relationships that could be construed as a potential conflict of interest.

## Publisher’s Note

All claims expressed in this article are solely those of the authors and do not necessarily represent those of their affiliated organizations, or those of the publisher, the editors and the reviewers. Any product that may be evaluated in this article, or claim that may be made by its manufacturer, is not guaranteed or endorsed by the publisher.

## Supplementary Material

Supplementary Table 1 Attention allocation studies organized by immersion level sections.
