# Supplementary material for: Attention Detection in Virtual Environments Using EEG Signals: A Scoping Review
Source: Front Physiol. 2021 Nov 23;12:727840. doi: 10.3389/fphys.2021.727840 (PMC8650681; doi:10.3389/fphys.2021.727840)
Supplement: Supplementary Table 1 — Attention allocation studies organized by immersion level sections. [file Table_1.docx]

| Author | Subjects (f+m) | Stimulus ( duration and times) | Main investigation through the eeg signal | Device | Sampling frequency | No. and electrodes location | Communication | Analytical method / classifier eeg signal | Artifact filtering | Eeg features | Feature extraction | Off vs on / user |
| --- | --- | --- | --- | --- | --- | --- | --- | --- | --- | --- | --- | --- |
| Engagement level of immersion | | | | | | | | | | | | |
| (Wang *et al.*, 2020) | 5+15 | 600s x 4 | AA | Neuroelectrics Enobio | 500 Hz | 32: All areas | NW | ICA | bandpass filter (1–120 Hz), downsampling to 250 Hz | δ, θ, α, β and γ | Short-time Fourier transform (STFT); PSD | OFF / UD |
| (Fuentes-García *et al.*, 2019) | 27 | 180s | VRV | MP150 - EEG100C | 250 Hz | 13 (F, C, P, O) | W | EEGlab (Matlab) | bandpass filter (0.5–35 Hz); ICA | θ | PSD; Statistical Analysis | OFF / UD |
| (Coenen *et al.*, 2020) | 0+14 | 600s x 10 | VRV | MindWAve/ BioSemi | 512Hz / 2048 Hz | 1 (Fp1) / 64 (Full cap) | Both | Statistical Analysis | downsampling to 250 Hz | Max amplitude and power: δ, θ, α, β and γ | FFT | OFF / UD |
| (Leite *et al.*, 2018) | 8+22 | 2s x 60 | VRV | g.SAHARAsys and g.USBamp | 256 Hz | 16 (O, P, C and FCz) | W | Linear classification | bandpass filter (5–60 Hz) of order 8; CAR | four peaks: 6, 10, 12, and 15 Hz | FFT, Statistical Analysis | OFF / UD |
| (Hazarika *et al.*, 2018) | 0+35 | 3s x 30 | AA | BIOPAC, MOBITA | 250 Hz | 32 (F, C, P, O) | NW | SVM, Wavelet-based | DWT, universal threshold and mean square error (MSE) | δ, θ, α, β and γ | Wavelet Energy variables, Statistical Analysis | OFF / UD |
| (C. Yang *et al.*, 2018) | 10 | 600s x 3 | AA | Neuroscan | 500 Hz | 27: All areas | W | CSP (common spatial pattern); LDA | bandpass filter (0.5–40 Hz) | SMR; TBR (θ/β ratio) | ERS; ERD | BOTH/ UD |
| (Touryan *et al.*, 2016) | 15+12 | 3000s | Drive simulation (fatigue) | BioSemi Active Two system | 1024 Hz | 256: Full cap | W | SVM, ICA, SFFS (Sequential Forward Floating Selection) | bandpass filter (1–30 Hz) | IC power spectra | FFT, PSD | OFF / BOTH |
| (Causse, Peysakhovich and Fabre, 2016) | 24 | 2400s | Drive Simulation (Workload) | Biosemi ActiveTwo | 512 Hz | 30 ( F, FP, AF, C, P, T, O) | W | ERP; ANOVA | bandpass filter (0.1-40 Hz) | MMN/ P3a, P3b, N400 and P600 components | Statistical Analysis | OFF / UD |
| (Asensio-Cubero, Gan and Palaniappan, 2016) | 7+7 | 8s x 40 | VRV | Biosemi ActiveTwo | 256 Hz | 15 ( F, C, FC, FP) | W | LDA; SFFS (sequential floating forward search) | bandpass flter (8–30 Hz) | graph lifting transform segments | CSP, Statistical Analysis | ON / UIN |
| (Lamti *et al.*, 2016) | 10 | 350s x 36 | Fatigue | Emotiv EPOC | 128 Hz | 14 (F, P, T, O) | NW | MLP (Multi Layer Perceptron), LDA and SVM | N/A | P300, N1, P2, θ, and α | Statistical Analysis | OFF / UD |
| (Yu *et al.*, 2015) | 16 | 600s x 4 | Workload | ANT amplifier | 512 Hz | 64: Full cap | W | SVM | bandpass filter (0.3–40 Hz) | P300 | PSD, BCSP, Statistical Analysis | OFF / UD |
| (Lee, Li and Fan, 2015) | 7+11 | 3600s | AA | Astro-Med Model QP511 Grass-Telefactor | 1000 Hz | 6 (Cz, C3, and C4 electrode pairs) | W | Linear classification | bandpass filter (0.1–100 Hz) | event-related desynchronization (ERD) | PSD, Statistical Analysis | OFF / UD |
| (Wang, Jung and Lin, 2015) | 0+10 | 900s x 25 | Drive simulation (attention) | Neuroscan | 500 Hz | 27: All areas | W | SVM with radial basis function (SVMRBF) | ICA | δ, θ, α and β | Stepwise Linear discriminant analysis (SWLDA); Statistical Analysis | OFF / UD |
| (Myrden and Chau, 2015) | 10+2 | 3000s | Fatigue and AA | B-Alert X24 | 256 Hz | 20 (F, C, P) | NW | LDA; PCA; Fast correlation-based filter (FCBF) | ICA; bandpass filter (2–30 Hz) | Spectral power within the signal in 1-Hz increments (from 0–1 to 29–30 Hz) | Fourier coefficients; Statistical Analysis; covariance matrix | BOTH / UD |
| (Rohani and Puthusserypady, 2015) | 1+5 | 1.5s x 12 | AA (Education) | G.USBamp | 256 Hz | 4 | W | SVM and Multiple Comparisons Problem (MCP) corrected permutation test | bandpass (0.5 – 30 Hz), low-pass filter and cut-off frequency of 12.8 Hz | P300 | N/A | OFF / UD |
| (Jagannath and Balasubramanian, 2014) | 0+20 | 3600s | Drive simulation (fatigue) | g Mitsar-EEG201 | 500 Hz | 21 (F, P, T, O) | W | WT (wavelet transformation coefficient energy) | bandpass filter (0.5–30 Hz) | θ, α, β; ratio (α+θ)/β; wavelet coefficient energy indices | Wavelet packet decomposition analysis; Statistical | OFF / UD |
| (Yin and Zhang, 2014) | 6 | 600s x 5 | Drive simulation (Workload) | Nihon Kohden | 500 Hz | 15 (AFz, F, P, T, O) | W | LLE-SVC; LLE-SVC-SVDD; LLE-SVDD; K-nearest neighbors | LLE; low-pass filter 40 Hz | frequency EEG features 1-40 Hz; θ, α, β and γ | PSD | OFF / UIN |
| (Cowley and Ravaja, 2014) | 15+20 | 3600s | VRV and learning | Becker, Meditech, Varioport-ARM | 1000 Hz | 6 (F3, F4, C3, C4, P3 and P4) | W | Brain Vision Analyser (Software) | FFT; power regression | δ, θ, α, β and γ; frontal asymmetry of EEG | Statistical Analysis | OFF |
| (Savage, Potter and Tatler, 2013) | 9+8 | 600s | Workload | BioSemi CHA-01 | 2500 Hz | 32: All areas | W | Statistical analysis | bandpass filter (3–10 Hz) | RT; θ | FFT | OFF |
| (Mathewson *et al.*, 2012) | 27+12 | 7200s x 10 | VRV, learning and workload | ElectroCap International | 100 Hz | 64: Full cap | W | Wavelet analysis | bandpass filter (0.01–30 Hz) | α and δ; P300 | ERSP; ERP | OFF |
| Engrossment level of immersion | | | | | | | | | | | | |
| (Lim, Yeo and Yoon, 2019) | 3+29 | 300s x 2 | AA | QEEG-8TM | 512 Hz | 8 (F, C, T, O) | W | ICA | bandpass filter (0.6–46 Hz) | Brain waves; θ/α bands index; β/θ ratio | Statistical Analysis | OFF / UD |
| (Petras, Oever and Jansma, 2016) | 20+10 | 6s x 400 | AA | EasyCap: 28 | 250 Hz | 28 ( F, C, P, O) | W | Brain Vision Analyser, FIeldTrip tools | bandpass filter (0.53–20 Hz) | N1, N2, P2 and P3 | Statistical Analysis (ANOVA) | OFF / UD |
| (Vogt *et al.*, 2015) | 10+12 | 300s x 2 | VRV | Brain Vision, Brain Products GmbH | 500 Hz | 64: Full cap | W | Statistical Analysis (ANOVA) | bandpass filter (0.5–50 Hz) | N200; P300 | Statistical Analysis | OFF / UD |
| (Matthews *et al.*, 2015) | 65+85 | 3600s | Workload | B-Alert X10 | 256 Hz | 9 (Fz, F3, F4, Cz, C3, C4, Pz, P3, and P4) | NW | Statistical Analysis (ANOVA) | High-pass and median filters were applied as well as 50, 60, 100, and 120 Hz notch filters | θ, α and β | PSD | OFF / UD |
| (Clemente *et al.*, 2014) | 9+11 | N/A | VRV | Emotiv EPOC | 128 Hz | 14 (F, P, T, O) | NW | sLORETA tool (standardized low-resolution electromagnetic tomography) | bandpass filter (0.5–45 Hz) | θ, α | Vowel-wise t-test | OFF / UD |
| (Chen *et al.*, 2014) | 0+10 | 4s x 480 | VRV | QuickAmp, Brain Products | 250 Hz | 30: All areas | W | DCM (bayesian model selection) | low-pass filter 30 Hz | SEP (P50, N80, P200); P300 | ANOVA, statistical tests, DCM (Dynamic Causal Modelling) | OFF / UD |
| (Wang, Sourina and Nguyen, 2011) | 5 | N/A | VRV | Emotiv EPOC | 128 Hz | 14 (F, P, T, O) | NW | Fractal dimension model | bandpass filter (2–42 Hz) | θ and β bands; θ/β ratio. | PSD | ON / UIN |
| Total immersion level | | | | | | | | | | | | |
| (Li, Zhou, *et al.*, 2020) | 13+17 | 1800s | AA | StarStim 8™ | 500 Hz | 8 (F, C, P, O) | NW | ICA and Linear classification | bandpass filter (4–30 Hz) | P3b; RT; ITC(θ) | Statistical Analysis | ON / UIN |
| (Li, Anguera, *et al.*, 2020) | 8+32 | 1980s | AA and VRV | Neuroelectrics Enobio | 500 Hz | 20 (F, C, P, T, O) | NW | ICA | bandpass filter (0.5–30 Hz) | ERP, ERSP, θ, α, β, P300, IEC, BTR (β/θ ratio) | Statistical Analysis | OFF / UD |
| (Vortmann, Kroll and Putze, 2019) | 3+12 | 13s x 24 | AA | g.Nautilus | 500 Hz | 16 (F, C, P, Oz) | W | LDA | ICA; bandpass filter (1–50 Hz); notch 50 Hz | θ, α, β and γ | PSD; Statistical Analysis | OFF / UD |
| (Gao *et al.*, 2019) | 62+58 | 300s | VRV, fatigue and AA | Neurosky Brainwave | 128 Hz | 1 (Fp1) | NW | Statistical Analysis | N/A | Attention and meditation levels | N/A | OFF / UD |
| (Magosso *et al.*, 2019) | 10+20 | 900s x 2 | Workload | OpenBCI Cyton Board + Daisy Board | 125 Hz | 12 (F, C, P, T, O) | W | ICA | highpass filter (0.75 Hz), 50 Hz notch filter | α | ANOVA, PSD | OFF / UD |
| (Heyselaar *et al.*, 2018) | 28+2 | 720s | AA | ActiCAP, Brainproducts | 500 Hz | 64: Full cap | W | linear mixed effects model | low-pass filter 150 Hz | δ, θ, α and β | FFT, Statistical Analysis | OFF / UD |
| (Peterson, Furuichi and Ferris, 2018) | 9+10 | 900s x 2 | Fatigue and workload | BioSemi Active II system | 512 Hz | 102: Full cap | W | K-means classification and clustering technique | ICA (power spectra and dipole locatin); high-pass filter 1 Hz | Dipole locations and IC clusters | FFT; Statistical Analysis | OFF / UD |
| (X. Yang *et al.*, 2018) | 16+14 | 300s | VRV, learning and AA | Neurosky Brainwave | 128 Hz | 1 (Fp1) | NW | Statistical Analysis | N/A | Attention and meditation levels | N/A | OFF / UD |
| (Chung, Lee and Park, 2018) | 22+18 | 300s | AA | EEG System (GES) 400 | 1000 Hz | 64: Full cap | W | ERP; RM-ANOVA | bandpass filter (0.3–30 Hz) | MMN/P3a | Statistical Analysis | OFF / UD |
| (Pereira *et al.*, 2018) | 9 | 1800s | VRV | Biosemi ActiveTwo amplifier | 2048 Hz | 30 (F, C, P, T, O) | W | N/A | Downsampling to 512 Hz and referenced to a common average | ERD | PSD, FFT, Statistical Analysis | OFF / UD |
| (Chu and D’zmura, 2019) | 8+12 | 10s x 128 | AA | WaveGuard ANT Neuro | 1024 Hz | 64: Full cap | W | FFT, LDA, SVM, Naïve Bayes (NB) | bandpass filter (1–50 Hz) | SSVEP (attend blue at 12.5 Hz, attend blue at 18.75 Hz) | SPD, FFT | OFF / UD |
| (Berger and Davelaar, 2018) | 8+14 | 2100s x 5 + 1800s | Learning, Workload and VRV | MyndPlay | 512 Hz | 2 (Fp1 and Fp2) | NW | Statistical Analysis (ANOVA) | baseline threshold | α and RT | FFT | OFF / UD |
| (Jaquess *et al.*, 2017) | 7+56 | 600s | Drive simulation (Workload and AA) | g.SAHARAsys and g.USBamp | 512 Hz | 4 (Fz, FCz, Cz, Pz) | W | ANOVA; canonical correlation | 50Hz low pass filter. Bandpass filter (0.01–40 Hz) | N1, P2, P3. θ, α and β | FFT; Statistical Analysis | OFF / UD |
| N/A: Not Available. VRV: Virtual Reality Variables. AA: Attention Allocation. W: Wired Communication. NW: Wireless Communication. LLE: Locally Linear Enbedding. FFT: Fast Fourier Transform. LDA: Linear Discriminant Analysis. ICA: Independent Component Analysis. SVC: Support Vector Clustering. SVDD: Support Vector Data Description. MWL: Mental Workload. UD: User-dependent data. UIN: User-independent data. MMN: Mismatch Negativity. ERP: Event-Related Potential. ERSP: Event-Related Spectral Perturbation. RT: Response Time. CSP: Common Spatial Patterns. | | | | | | | | | | | | |
